# 2-Meth­oxy-4-[(4-methyl­piperazin-1-yl)imino­meth­yl]phenol

**DOI:** 10.1107/S1600536810051135

**Published:** 2010-12-11

**Authors:** Li-Na Zhou, Long Yan, Hui-Liang Zhou, Qing-Feng Yang, Qi-Lin Hu

**Affiliations:** a College of Chemistry and Chemical Engineering, Ningxia University, Yinchuan 750021, Ninxia, People’s Republic of China; b Key Laboratory of Energy Resource and Chemical Engineering, Yinchuan 750021, Ninxia, People’s Republic of China

## Abstract

The title compound, C_13_H_19_N_3_O_2_, was obtained by the direct solvent-free reaction of 4-hy­droxy-3-meth­oxy­benzaldehyde with 1-amino-4-methyl­piperazine. The piperazine ring adopts a chair conformation. In the crystal, strong inter­molecular O—H⋯N and weak inter­molecular C—H⋯O and C—H⋯N hydrogen bonds help to establish the packing.

## Related literature

For the biological properties of piperazine compounds, see: Obniska *et al.* (2005 ▶); Smid *et al.* (2005 ▶). For background and related structures, see: Guo (2004 ▶, 2007 ▶).
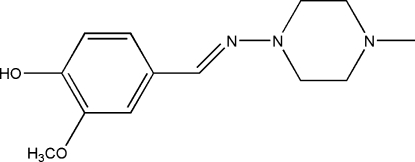


## Experimental

### 

#### Crystal data

C_13_H_19_N_3_O_2_
*M* *_r_* = 249.31
Orthorhombic, 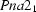

*a* = 12.179 (2) Å
*b* = 18.624 (3) Å
*c* = 6.0187 (10) Å
*V* = 1365.1 (4) Å^3^
*Z* = 4
Mo *K*α radiation
μ = 0.08 mm^−1^
*T* = 296 K
0.25 × 0.25 × 0.10 mm

#### Data collection

Siemens SMART CCD diffractometer
Absorption correction: multi-scan (*SADABS*; Bruker, 2002 ▶) *T*_min_ = 0.642, *T*_max_ = 0.745
7503 measured reflections
1582 independent reflections
1126 reflections with *I* > 2σ(*I*)
*R*_int_ = 0.043

#### Refinement

*R*[*F* ^2^ > 2σ(*F* ^2^)] = 0.040
*wR*(*F* ^2^) = 0.077
*S* = 1.01
1582 reflections
169 parameters
1 restraint
H atoms treated by a mixture of independent and constrained refinement
Δρ_max_ = 0.13 e Å^−3^
Δρ_min_ = −0.13 e Å^−3^

### 

Data collection: *SMART* (Bruker, 2007 ▶); cell refinement: *SAINT* (Bruker, 2007 ▶); data reduction: *SAINT*; program(s) used to solve structure: *SHELXS97* (Sheldrick, 2008 ▶); program(s) used to refine structure: *SHELXL97* (Sheldrick, 2008 ▶); molecular graphics: *DIAMOND* (Brandenburg, 1999) ▶; software used to prepare material for publication: *SHELXL97*.

## Supplementary Material

Crystal structure: contains datablocks I, global. DOI: 10.1107/S1600536810051135/im2254sup1.cif

Structure factors: contains datablocks I. DOI: 10.1107/S1600536810051135/im2254Isup2.hkl

Additional supplementary materials:  crystallographic information; 3D view; checkCIF report

## Figures and Tables

**Table 1:** Hydrogen-bond geometry (Å, °)

*D*—H⋯*A*	*D*—H	H⋯*A*	*D*⋯*A*	*D*—H⋯*A*
O1—H1⋯N2^i^	0.94 (4)	1.88 (4)	2.734 (3)	151 (3)
C11—H11*A*⋯O2^ii^	0.96	2.67	3.311 (4)	125
C5—H5⋯N1^iii^	0.93	2.67	3.460 (4)	143

Symmetry codes: (i) 
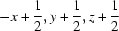
; (ii) 
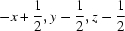
; (iii) 
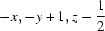
.

**Fig. 1.**
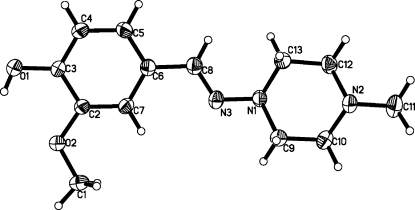
Molecular structure of the title compound, showing 30% displacement ellipsoids for non-hydrogen atoms. Hydrogen atoms are drawn as spheres of arbitrary radius.

**Fig. 2.**
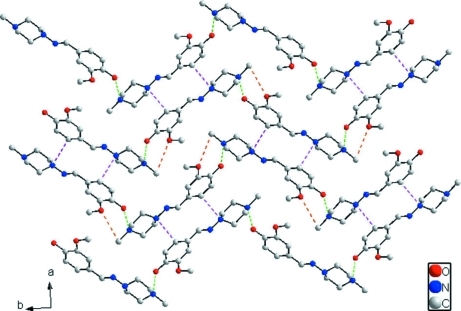
Molecular packing of the title compound, viewed along the c axis. Hydrogen bonds are shown as dashed lines.

## supplementary crystallographic information

### Comment

Piperazine and it's derivatives are important targets for drug discovery. For the biological properties of piperazine compounds, see: Obniska *et al.* (2005); Smid *et al.* (2005). For background of this study and related structures, see: Guo (2004); Guo (2007).

The title compound, (I), is a hydrazone in which 4-hydroxy-3-methoxy-benzaldehyde has reacted directly with 1-amino-4-methylpiperazine to form a product containing the C=N double bond. The structure of the compound is shown in Fig. 1. The C=N double bond shows an *E* configuration and is effectively coplanar with the benzene ring [N3–C8–C6–C7=1.6 (5)°]. The piperazine ring exhibits a chair conformation. The bond distances and angles are normal. In the crystal structure, strong intermolecular N—H···O and weak intermolecular C—H···O and C—H···N hydrogen bonds (see Table 1 for symmetry code) and van der Waals forces are responsible for the observed packing motif. A packing diagram for (I) is shown in Fig. 2.

### Experimental

The title compound was prepared by the direct solvent-free reaction of 4-hydroxy-3-methoxy-benzaldehyde (1.52 g) with 1-amino-4-methylpiperazine (1.15 g) with stirring at 351 K for 30 min. The resulting product was dissolved in ethanol (10 ml) with heating. The homogeneous solution was allowed to stand at room temperature for 12 h, after which the crystalline product was separated by filtration (yield 2.0 g, 80%). The pure product (0.5 g) was dissolved in hot ethanol (20 ml). Single crystals were obtained from this solution by slow evaporation over a period of 7 d at room temperature.

### Refinement

In the absence of significant anomalous dispersion effects Friedel pairs have been merged. All H atoms were positioned geometrically and refined using the riding-model approximation, with C—H = 0.93 or 0.96 Å, O—H = 0.82 Å, N—H = 0.86 Å and *U*_iso_(H) = 1.2*U*_eq_(C, N) or *U*_iso_(H) = 1.5*U*_eq_(methyl C and O).

### Crystal data

**Table:**

C_13_H_19_N_3_O_2_	*D*_x_ = 1.213 Mg m^−^^3^
*M**_r_* = 249.31	Melting point: not measured K
Orthorhombic, *P**n**a*2_1_	Mo *K*α radiation, λ = 0.71073 Å
Hall symbol: P 2c -2n	Cell parameters from 1708 reflections
*a* = 12.179 (2) Å	θ = 2.0–25.1°
*b* = 18.624 (3) Å	µ = 0.08 mm^−^^1^
*c* = 6.0187 (10) Å	*T* = 296 K
*V* = 1365.1 (4) Å^3^	Club-shaped, colorless
*Z* = 4	0.25 × 0.25 × 0.10 mm
*F*(000) = 536	

### Data collection

**Table:**

Siemens SMART CCD diffractometer	1582 independent reflections
Radiation source: fine-focus sealed tube	1126 reflections with *I* > 2σ(*I*)
graphite	*R*_int_ = 0.043
Detector resolution: 9.00 cm pixels mm^-1^	θ_max_ = 26.7°, θ_min_ = 2.0°
ω scans	*h* = −15→14
Absorption correction: multi-scan (*SADABS*; Bruker, 2002)	*k* = −23→21
*T*_min_ = 0.642, *T*_max_ = 0.745	*l* = −7→6
7503 measured reflections	

### Refinement

**Table:**

Refinement on *F*^2^	Secondary atom site location: difference Fourier map
Least-squares matrix: full	Hydrogen site location: inferred from neighbouring sites
*R*[*F*^2^ > 2σ(*F*^2^)] = 0.040	H atoms treated by a mixture of independent and constrained refinement
*wR*(*F*^2^) = 0.077	*w* = 1/[σ^2^(*F*_o_^2^) + (0.010*P*)^2^ + 0.480*P*] where *P* = (*F*_o_^2^ + 2*F*_c_^2^)/3
*S* = 1.01	(Δ/σ)_max_ < 0.001
1582 reflections	Δρ_max_ = 0.13 e Å^−^^3^
169 parameters	Δρ_min_ = −0.13 e Å^−^^3^
1 restraint	Extinction correction: *SHELXL97* (Sheldrick, 2008), Fc^*^=kFc[1+0.001xFc^2^λ^3^/sin(2θ)]^-1/4^
Primary atom site location: structure-invariant direct methods	Extinction coefficient: 0.0157 (12)

### Special details

**Table:**

Geometry. All e.s.d.'s (except the e.s.d. in the dihedral angle between two l.s. planes) are estimated using the full covariance matrix. The cell e.s.d.'s are taken into account individually in the estimation of e.s.d.'s in distances, angles and torsion angles; correlations between e.s.d.'s in cell parameters are only used when they are defined by crystal symmetry. An approximate (isotropic) treatment of cell e.s.d.'s is used for estimating e.s.d.'s involving l.s. planes.
Refinement. Refinement of *F*^2^ against ALL reflections. The weighted *R*-factor *wR* and goodness of fit *S* are based on *F*^2^, conventional *R*-factors *R* are based on *F*, with *F* set to zero for negative *F*^2^. The threshold expression of *F*^2^ > σ(*F*^2^) is used only for calculating *R*-factors(gt) *etc*. and is not relevant to the choice of reflections for refinement. *R*-factors based on *F*^2^ are statistically about twice as large as those based on *F*, and *R*- factors based on ALL data will be even larger.

### Fractional atomic coordinates and isotropic or equivalent isotropic displacement parameters (Å^2^)

**Table:**

	*x*	*y*	*z*	*U*_iso_*/*U*_eq_	
O1	0.38591 (18)	0.68018 (10)	0.1639 (4)	0.0597 (7)	
H1	0.440 (3)	0.6712 (18)	0.271 (7)	0.090*	
O2	0.39131 (16)	0.56755 (10)	0.4468 (4)	0.0563 (6)	
N1	0.02060 (18)	0.33961 (11)	0.1024 (4)	0.0441 (6)	
N2	−0.05455 (17)	0.20217 (11)	−0.0440 (5)	0.0459 (6)	
N3	0.09346 (17)	0.39555 (11)	0.1421 (5)	0.0460 (6)	
C1	0.3972 (3)	0.50737 (15)	0.5919 (6)	0.0621 (9)	
H1A	0.3291	0.5025	0.6707	0.093*	
H1B	0.4560	0.5143	0.6961	0.093*	
H1C	0.4107	0.4647	0.5068	0.093*	
C2	0.3147 (2)	0.56473 (14)	0.2784 (5)	0.0406 (7)	
C3	0.3161 (2)	0.62428 (13)	0.1367 (5)	0.0433 (7)	
C4	0.2429 (2)	0.62631 (14)	−0.0381 (6)	0.0500 (7)	
H4	0.2428	0.6656	−0.1336	0.060*	
C5	0.1695 (2)	0.57068 (14)	−0.0731 (6)	0.0491 (8)	
H5	0.1202	0.5733	−0.1908	0.059*	
C6	0.1687 (2)	0.51122 (14)	0.0650 (5)	0.0429 (7)	
C7	0.2418 (2)	0.50933 (14)	0.2442 (5)	0.0439 (7)	
H7	0.2412	0.4704	0.3408	0.053*	
C8	0.0937 (2)	0.45134 (14)	0.0184 (6)	0.0478 (8)	
H8A	0.0471	0.4551	−0.1155	0.057*	
C9	0.0658 (2)	0.27338 (13)	0.1924 (6)	0.0517 (8)	
H9A	0.0887	0.2812	0.3449	0.062*	
H9B	0.1299	0.2594	0.1070	0.062*	
C10	−0.0186 (2)	0.21406 (15)	0.1845 (6)	0.0518 (8)	
H10A	0.0129	0.1702	0.2435	0.062*	
H10B	−0.0812	0.2270	0.2759	0.062*	
C11	−0.1364 (2)	0.14440 (14)	−0.0515 (7)	0.0655 (10)	
H11A	−0.1034	0.1003	−0.0032	0.098*	
H11B	−0.1628	0.1390	−0.2009	0.098*	
H11C	−0.1967	0.1562	0.0446	0.098*	
C12	−0.1020 (2)	0.26857 (13)	−0.1319 (6)	0.0524 (9)	
H12A	−0.1667	0.2810	−0.0461	0.063*	
H12B	−0.1247	0.2609	−0.2845	0.063*	
C13	−0.0210 (2)	0.33023 (15)	−0.1233 (6)	0.0500 (9)	
H13A	0.0398	0.3206	−0.2233	0.060*	
H13B	−0.0567	0.3741	−0.1718	0.060*	

### Atomic displacement parameters (Å^2^)

**Table:**

	*U*^11^	*U*^22^	*U*^33^	*U*^12^	*U*^13^	*U*^23^
O1	0.0704 (15)	0.0501 (12)	0.0588 (16)	−0.0206 (11)	−0.0153 (13)	0.0126 (12)
O2	0.0664 (13)	0.0492 (11)	0.0535 (14)	−0.0121 (10)	−0.0184 (14)	0.0125 (12)
N1	0.0432 (13)	0.0371 (12)	0.0519 (17)	−0.0026 (10)	−0.0032 (13)	−0.0023 (12)
N2	0.0443 (12)	0.0383 (12)	0.0550 (17)	−0.0042 (10)	0.0015 (14)	−0.0058 (14)
N3	0.0453 (13)	0.0377 (12)	0.0549 (17)	−0.0026 (11)	0.0025 (13)	−0.0054 (13)
C1	0.075 (2)	0.0572 (18)	0.054 (2)	0.0014 (16)	−0.013 (2)	0.0147 (18)
C2	0.0438 (16)	0.0369 (15)	0.0410 (17)	0.0015 (12)	−0.0031 (14)	0.0023 (14)
C3	0.0462 (16)	0.0365 (15)	0.0473 (19)	−0.0047 (12)	−0.0008 (15)	0.0041 (16)
C4	0.0592 (18)	0.0406 (15)	0.0502 (19)	−0.0013 (14)	−0.0062 (18)	0.0078 (17)
C5	0.0479 (16)	0.0477 (16)	0.052 (2)	0.0020 (14)	−0.0128 (17)	0.0035 (16)
C6	0.0377 (15)	0.0378 (14)	0.053 (2)	0.0014 (12)	−0.0004 (14)	−0.0016 (14)
C7	0.0460 (16)	0.0352 (14)	0.051 (2)	−0.0021 (13)	−0.0013 (16)	0.0065 (14)
C8	0.0420 (16)	0.0428 (15)	0.059 (2)	0.0007 (13)	−0.0032 (15)	−0.0021 (15)
C9	0.0599 (18)	0.0465 (16)	0.049 (2)	−0.0022 (14)	−0.0068 (16)	0.0026 (16)
C10	0.0581 (19)	0.0421 (16)	0.055 (2)	−0.0050 (14)	0.0064 (17)	0.0006 (16)
C11	0.0586 (19)	0.0456 (16)	0.092 (3)	−0.0088 (14)	0.001 (2)	−0.008 (2)
C12	0.0487 (17)	0.0434 (16)	0.065 (2)	0.0024 (14)	−0.0094 (15)	−0.0078 (16)
C13	0.0531 (18)	0.0421 (17)	0.055 (2)	0.0010 (14)	−0.0105 (16)	0.0010 (15)

### Geometric parameters (Å, °)

**Table:**

O1—C3	1.354 (3)	C5—C6	1.385 (4)
O1—H1	0.94 (4)	C5—H5	0.9300
O2—C2	1.378 (3)	C6—C7	1.399 (4)
O2—C1	1.423 (3)	C6—C8	1.468 (4)
N1—N3	1.389 (3)	C7—H7	0.9300
N1—C9	1.456 (3)	C8—H8A	0.9884
N1—C13	1.460 (4)	C9—C10	1.510 (4)
N2—C10	1.460 (4)	C9—H9A	0.9700
N2—C12	1.464 (3)	C9—H9B	0.9700
N2—C11	1.468 (3)	C10—H10A	0.9700
N3—C8	1.278 (3)	C10—H10B	0.9700
C1—H1A	0.9600	C11—H11A	0.9600
C1—H1B	0.9600	C11—H11B	0.9600
C1—H1C	0.9600	C11—H11C	0.9600
C2—C7	1.377 (4)	C12—C13	1.515 (4)
C2—C3	1.399 (4)	C12—H12A	0.9700
C3—C4	1.380 (4)	C12—H12B	0.9700
C4—C5	1.384 (3)	C13—H13A	0.9700
C4—H4	0.9300	C13—H13B	0.9700
			
C3—O1—H1	113 (2)	N3—C8—C6	120.5 (3)
C2—O2—C1	117.1 (2)	N3—C8—H8A	122.1
N3—N1—C9	109.2 (2)	C6—C8—H8A	117.3
N3—N1—C13	118.1 (2)	N1—C9—C10	110.5 (2)
C9—N1—C13	112.1 (2)	N1—C9—H9A	109.5
C10—N2—C12	109.3 (2)	C10—C9—H9A	109.5
C10—N2—C11	110.1 (3)	N1—C9—H9B	109.5
C12—N2—C11	109.9 (2)	C10—C9—H9B	109.5
C8—N3—N1	120.7 (2)	H9A—C9—H9B	108.1
O2—C1—H1A	109.5	N2—C10—C9	110.2 (3)
O2—C1—H1B	109.5	N2—C10—H10A	109.6
H1A—C1—H1B	109.5	C9—C10—H10A	109.6
O2—C1—H1C	109.5	N2—C10—H10B	109.6
H1A—C1—H1C	109.5	C9—C10—H10B	109.6
H1B—C1—H1C	109.5	H10A—C10—H10B	108.1
C7—C2—O2	125.1 (3)	N2—C11—H11A	109.5
C7—C2—C3	120.7 (3)	N2—C11—H11B	109.5
O2—C2—C3	114.2 (2)	H11A—C11—H11B	109.5
O1—C3—C4	118.5 (3)	N2—C11—H11C	109.5
O1—C3—C2	122.9 (3)	H11A—C11—H11C	109.5
C4—C3—C2	118.6 (2)	H11B—C11—H11C	109.5
C3—C4—C5	120.9 (3)	N2—C12—C13	111.8 (2)
C3—C4—H4	119.6	N2—C12—H12A	109.3
C5—C4—H4	119.6	C13—C12—H12A	109.3
C4—C5—C6	120.8 (3)	N2—C12—H12B	109.3
C4—C5—H5	119.6	C13—C12—H12B	109.3
C6—C5—H5	119.6	H12A—C12—H12B	107.9
C5—C6—C7	118.6 (3)	N1—C13—C12	110.4 (3)
C5—C6—C8	119.9 (3)	N1—C13—H13A	109.6
C7—C6—C8	121.6 (3)	C12—C13—H13A	109.6
C2—C7—C6	120.5 (3)	N1—C13—H13B	109.6
C2—C7—H7	119.8	C12—C13—H13B	109.6
C6—C7—H7	119.8	H13A—C13—H13B	108.1
			
C9—N1—N3—C8	155.1 (3)	C5—C6—C7—C2	−1.6 (4)
C13—N1—N3—C8	25.5 (4)	C8—C6—C7—C2	177.0 (3)
C1—O2—C2—C7	2.0 (4)	N1—N3—C8—C6	178.2 (2)
C1—O2—C2—C3	−177.6 (3)	C5—C6—C8—N3	178.7 (3)
C7—C2—C3—O1	179.7 (3)	C7—C6—C8—N3	0.1 (4)
O2—C2—C3—O1	−0.7 (4)	N3—N1—C9—C10	171.1 (3)
C7—C2—C3—C4	0.0 (4)	C13—N1—C9—C10	−56.0 (3)
O2—C2—C3—C4	179.6 (3)	C12—N2—C10—C9	−59.3 (3)
O1—C3—C4—C5	−179.8 (3)	C11—N2—C10—C9	179.9 (2)
C2—C3—C4—C5	−0.1 (4)	N1—C9—C10—N2	58.7 (3)
C3—C4—C5—C6	−0.7 (5)	C10—N2—C12—C13	58.0 (3)
C4—C5—C6—C7	1.5 (4)	C11—N2—C12—C13	178.9 (3)
C4—C5—C6—C8	−177.1 (3)	N3—N1—C13—C12	−177.9 (2)
O2—C2—C7—C6	−178.7 (3)	C9—N1—C13—C12	53.7 (3)
C3—C2—C7—C6	0.8 (4)	N2—C12—C13—N1	−54.9 (3)

### Hydrogen-bond geometry (Å, °)

**Table:**

*D*—H···*A*	*D*—H	H···*A*	*D*···*A*	*D*—H···*A*
O1—H1···N2^i^	0.94 (4)	1.88 (4)	2.734 (3)	151 (3)
C11—H11A···O2^ii^	0.96	2.67	3.311 (4)	125.
C5—H5···N1^iii^	0.93	2.67	3.460 (4)	143.

Symmetry codes: (i) −*x*+1/2, *y*+1/2, *z*+1/2; (ii) −*x*+1/2, *y*−1/2, *z*−1/2; (iii) −*x*, −*y*+1, *z*−1/2.
